# Farmers’ Willingness to Pay for Health Risk Reductions of Pesticide Use in China: A Contingent Valuation Study

**DOI:** 10.3390/ijerph15040625

**Published:** 2018-03-29

**Authors:** Wenyu Wang, Jianjun Jin, Rui He, Haozhou Gong, Yuhong Tian

**Affiliations:** 1 State Key Laboratory of Earth Surface Processes and Resource Ecology, Beijing Normal University, Beijing 100875, China; 201521190014@mail.bnu.edu.cn (W.W.); 201621190023@mail.bnu.edu.cn (R.H.); hzgong@163.com (H.G.); tianyuhong@bnu.edu.cn (Y.T.); 2 Faculty of Geographical Sciences, Beijing Normal University, Beijing 100875, China

**Keywords:** pesticides, contingent valuation method, willingness to pay, China

## Abstract

This study aimed to estimate farmers’ willingness to pay (WTP) for health risk reductions of pesticide use by applying the contingent valuation method (CVM) and to explore the factors that influence farmers’ WTP in China. In total, 244 farmers were randomly selected and interviewed. The mean WTP was estimated to be $65.38 (0.94% of total household income) per household per year for a 5/10,000 reduction in morbidity risk. This study shows that farmers’ socioeconomic and attitudinal factors that significantly affect their WTP include farmers’ farming income, education, household size and risk perceptions. In particular, the results demonstrate that respondents’ social trust, social reciprocity and social networks have significant impacts on their WTP. The findings of this study can provide useful insights for policy makers to design effective policies to address health problems related to pesticide use in the developing world.

## 1. Introduction

Pesticides have been playing a significant role in the success of modern agricultural production [1,2]. However, the intensive use of pesticides had adverse impacts on the environment and human health [3,4,5,6]. The use of pesticide poses morbidity and mortality risks to farmers and agricultural workers. It has been shown that pesticide exposure could result in various diseases such as cardiovascular disease, acute neurological toxicity, cancer, allergies and neurological disorders [7,8]. Farmers’ health status impairment may have a significant negative impact on agricultural production [9]. Pesticide poisoning has been a major health risk in developing countries, while chronic effects from long-term exposure to pesticides are not widely recognized and documented [10].

China is the largest consumer of pesticides in the world [11]. Local governments are making efforts to develop better policies regulating pesticide usage to minimize the hazards and risks to human health and the environment from the use of pesticides [12]. In order to design more effective pesticide regulation policies and to reduce pesticide poisoning among farmers, it is important to understand more about the monetary value of changing health risks related to pesticide use to perform an economic analysis [13,14,15]. Because the health costs of pesticide use include market and non-market components such as discomfort and pain, it is important to conduct a more comprehensive analysis that takes the non-market value of human health into account [9]. For this purpose, the contingent valuation method (CVM) is considered to be an appropriate method to value individuals’ willingness-to-pay (WTP) for health risk changes [10]. The basic idea of the CVM is that people’s preference for non-marketed goods or services can be revealed if appropriate questions are asked in a hypothetical market questionnaire survey [16]. This study is to estimate farmers’ WTP for health risk reductions of pesticide use by applying the CVM.

Furthermore, to help policymakers design more effective policies, understanding the factors influencing individuals’ preferences or willingness to pay is important. The environmental economics literature indicates that both socio-economic factors (i.e., age, education attainment, household income and household size) and pro-environmental attitudes (i.e., environmental awareness and perceptions) can affect peoples’ WTP for non-market goods or services (see for example [17,18]). In the past decades, the literature of environmental policy and management has successfully introduced the concept of social capital [19], which is a form of economic and cultural capital. Social capital captures the idea that social bonds and norms are important for people and communities, which has been found to have influences on individuals’ environmental behaviors [20,21]. Consequently, it is important to understand the influence of an individual’s social capital on his or her WTP for health improvements. In this context, another important objective of this study is to explore the factors that influence farmers’ WTP and look into an individual’s social capital on his WTP for reducing health risks of pesticide use in China. This study can provide useful insights for policy makers to design effective policies to address health problems associated with pesticide use in China and other similar developing countries.

## 2. Materials and Methods

### 2.1. The Study Area

The study area was Anqiu County (118°44′–119°27′ E and 36°05′–36°38′ N), which is located in the central part of Shandong Province, China. The total population is 950,000 inhabitants, among which the agricultural population accounted for 56%. The average population density is approximately 539 inhabitants/km^2^. Ten different crops (winter wheat, scallions, peanuts, garlic, gingers, corns, potatoes, nectarines, strawberries, cherries) are grown in the area. The average per capita GDP of agricultural population was CNY 13,575 (USD 1966). This particular area was chosen because of the fact that the county has been adopting high-input farming practices, including pesticide application. Based on the information provided by local agricultural management agencies and retailers the most commonly applied pesticides in Anqiu are insecticides (abamectin, fosthiazate, thiamethoxam, dinotefuran, chlorantraniliprole) and fungicides (metalaxyl, fludioxonil).

### 2.2. Survey Instrument

The survey questionnaire was carefully designed based on the results of several focus-group discussions and pilot testing surveys. Some local farmers, government officials and scientific experts were invited to attend focus-group discussions to obtain opinions on the research design and to assess the suitability of WTP bids used. Questionnaires were further pilot tested on local farmers to further identify potential problems. Based on the feedback of pilot testing surveys, we made some clarifications and modifications.

The questionnaire mainly consisted of four sections. The first section collected information on farmers’ knowledge and risk perceptions of pesticide use. The extent of respondents’ agreement or disagreement was elicited by a series of statements concerning pesticide use and its health effects on a five-point Likert scale anchored at 1 = Strongly disagree to 5 = Strongly agree. Respondents were also asked to indicate the risk of pesticide use on their health using the following five categories: extremely high level of risk, large (or significant) level of risk, medium (or moderate) level of risk, some small level of risk, and no risk at all.

The second part was the measurement of social capital. Based on social capital measurement studies (for example [20,22]), in this study, four components of social capital were emphasized: social trust, social networks, institutional trust and social reciprocity. Firstly, social trust was measured through farmers’ trust towards people in villages in general. Secondly, social networks were measured by the number of village associations that the respondent participated in. Institutional trust refers to the trust in institutions operating in the community [22]. Thus, trust in local governments was measured. Finally, social reciprocity was measured by asking the respondent’s agreement on the statement that people in their village help each other on a five-point Likert scale, going from 1 (strongly disagree) to 5 (strongly agree).

The third part evoked respondents’ willingness to pay for health risk reductions related to pesticide use. Respondents were first informed some basic information on the impact of pesticide use on human health. To get a point of reference, respondents were informed the number of yearly cases of hospitalization and death due to pesticide use in China. 

Because health risks associated with pesticide use are typically small, a risk scale with a graph containing 1000 squares was used to help respondents to understand. The use of a grid of squares has been considered a successful risk communication tool in other stated preference investigations involving health risk [23] and has been used in a developing country context [24,25].

Then a hypothetical valuation scenario was presented. Respondents were informed that implementing pesticide management policies can reduce health risks of pesticide use. Policy options, including agricultural production practices, are designed to reduce the rate of pesticide use in the fields, without any change in the quality of products. However, production costs would be increased due to the reduction of pesticide use. After this scenario, a dichotomous choice WTP question was presented. Respondents were asked whether their households would be willing to pay a specific amount annually to reduce the morbidity risk associated with pesticide use reduced by 5/10,000 per year. Six different bids were used: 200, 400, 600, 800, 1000 CNY (CNY is Chinese currency, 1 USD = 6.5 CNY). The bids were established base on the results from focus-group discussions and pilot testing surveys. For each respondent, a single randomly assigned dollar was presented. The last section collected socioeconomic information of the respondents including gender, age, farming income, formal education and years of farming experience.

### 2.3. Data Collection

This study used the stratified random sampling method to select respondents. Firstly, seven towns with agriculture production activities were selected from fourteen towns in Anqiu. Secondly, three villages were randomly chosen in each selected town. Finally, according to the parameters of the household characteristics (farm size, household size and income), 10–15 households were randomly selected from each village. The respondent in each household was the person who did most of the farm work and used pesticides. The reason why farmers were chosen as our respondents is that they are faced with the highest health risks related to pesticides [26]. Finally, a total of 261 farmers were approached and 244 participated in the study. The overall response rate was 94%.

The survey was conducted between July and August 2016 in Anqiu County. Face-to-face interviews were carried out to encourage more responses. This survey method is expensive but can provide the highest response rates and is better suited to collecting complex information [9].

### 2.4. Data Analysis

In this study, a binary logit regression model was used to analyze the data because it is preferred to the probit model in many fields due to its relative computational simplicity [27]. The basic relationship is given as:

$$P_{i}(yes)=\frac{1}{1+\exp[-(\alpha -\beta A)]}$$

where *α* and *β* are coefficients to be estimated using the maximum likelihood (ML) estimation method and *A* is the bid amount that the respondent was asked to pay. Based on the above equation, the mean WTP can be estimated [28]:

Mean WTP = −*α*/*β*

where *β* is the estimated coefficient on the bid amount and *α* is either the estimated constant or the grand constant calculated as the sum of the estimated constant plus the product of the coefficient estimates on other independent variables and times their respective means [29].

## 3. Results and Discussion

### 3.1. Socioeconomic Characteristics of the Respondents

The sample’s main socioeconomic characteristics is presented in Table 1. Most of the respondents were male farmers (*n* = 244, 69%). This result is realistic and understandable because men conduct most agricultural activities and take more responsibility for purchasing and spraying pesticides in the study area. The mean age of the respondents was 52 years old with a range from 30 to 86 years. The reason for this is due to the fact that many young farmers leave farms to look for better jobs in cities. The mean years of schooling were seven, close to middle school. Approximately 8% of the respondents were illiterate (data not shown). The average household income from farming (crops, vegetables, fruits and others) was 24,908 yuan/year (about $3617). Our results show that respondents’ socioeconomic characteristics were in line with the average values for the real population in the whole county, suggesting that our sample is representative.

### 3.2. Perceptions of Farmers on Pesticide Risk 

Most respondents strongly agreed (41.39%) or agreed (38.93%) with the statement that the use of pesticides in food production can reduce food safety. A large majority of the sample (86%) strongly agreed or agreed with the statement that pesticide use has adverse effects on human health. Approximately 26.23% of the respondents strongly agreed they could try to avoid the harm caused by pesticide exposure. Respondents were asked to assess their personal health risks associated with pesticide use. About 13.11% of the respondents reported that the risk of pesticide use on their health was extremely high, 27.05% thought the risk to be large, 34.43% believed the risk to be moderate or medium, 20.90% reported a low level of risk, and 4.51 percent stated that there was no risk at all when using pesticides.

### 3.3. Social Capital

Several questions intended to measure the respondents’ individual social capital, including social trust, social networks, social reciprocity and institutional trust. Approximately, 59% and 37% of respondents strongly agreed and agreed that people in their village helped each other. Our results also show that 49% of farmers in our study strongly agreed that people in their village could be trusted; 39% agreed with this view; only 10% and 2% of farmers were uncertain or disagreed. More than half of the respondents (63.93%) claimed that they were not a member of any village associations. 21.72% of the sample were members of village associations. Approximately, 16% of the respondents attended two or more associations. In total, 74% of the sample stated that they trusted local governments. 

### 3.4. Respondents’ Attitudes toward Payment

On the WTP question, 58.6% of the sample responded positively. They expressed that they would be willing to pay an amount to reduce their health risks. The rest 41.4% responded negatively. Respondents were asked to indicate their reasons for their responses to the WTP question. As shown in Table 2, the most important motivation for payment was the valuation program would be good for their health in the future. 37.82% of those willing to contribute believed that the program can reduce the morbidity of farmers.

According to the survey results (Table 3), 38.61% of the non-contributors stated that they cannot afford the payment. 35.64% stated that their reason for zero contribution was local governments should bear the costs. 18.81% of those who answered “no” claimed that they didn’t think they would get diseases because of pesticides and 4.95% declared that they didn’t believe the valuation program.

### 3.5. Farmers’ Willingness to Pay and Influencing Factors

**Figure 1:**
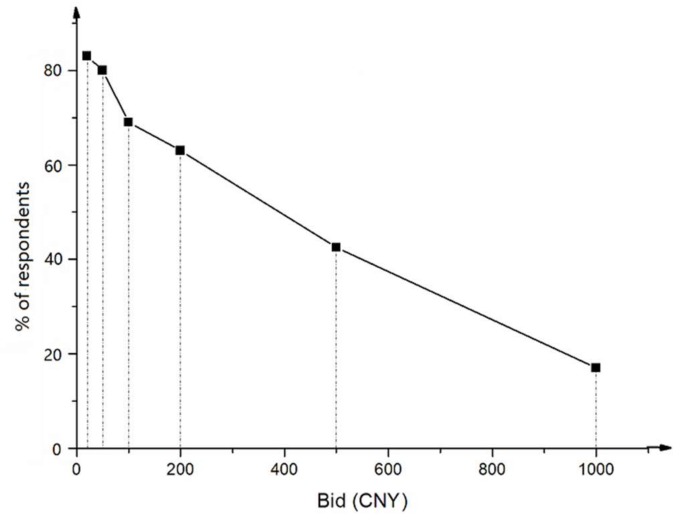
The percentage of “yes” responses to the WTP questions.

Figure 1 shows the percentage of “yes” responses to the WTP questions. It can be seen that the percentage of “yes” responses monotonically decreases as the bid amount goes up, implying that a higher bid would result in a lower probability of accepting the bid amount. This finding is consistent with the economic theory of demand, thus serving as evidence of the validity of this CVM study [16,30].

In order to identify the determinants influencing farmers’ WTP, we run the binary logit regression model. In the model, the dependent variable was the possibility that the respondent would be willing to pay. The explanatory variables included the bid amount, farmers risk perceptions, individual social capital variables and certain demographic and socioeconomic variables.

The estimation results are summarized in Table 4. Our results suggest that the estimation model has a high predictive power and statistical reliability. The pseudo-R^2^ in this model is higher than the 20% level suggested by [31] as indicating a very good fit for this type of data. The chi-square result shows that the likelihood ratio statistics are highly significant (*p* < 0.001), suggesting that the regression model has a strong explanatory power.

As shown in Table 4, most explanatory variables have the expected signs and are statistically significant at 10% or lower. As expected, the bid variable is negative and significant at the 1% level, suggesting that the respondents would have a lower probability to say “yes” to the WTP question if they were presented with a higher bid, in line with earlier findings in the survivor curve.

Considering farmers’ risk perceptions of pesticide use on their health, it comes as no surprise that the variable “risk”, indicating farmers’ risk perceptions, is positive and significant, implying that respondents with a higher risk perception would have a higher probability to be willing to pay for reducing their health risks associated with pesticide use. Khan and Damalas (2015) also argue that farmers who perceive pesticides as a health risk are more willing to pay for a premium relative to those who do not perceive pesticides as a health risk [9].

The estimation results show that farmers’ education level and household farming income have positive effects on their willingness to pay. These results are in accordance with empirical findings in earlier literature. The longer time to receive education, the better people understand the consequences of pesticide use on health and the need to reduce the health risks. Therefore, the educated will be more willing to pay the premium than the illiterate. Wohl et al. (1995) reported that the respondent with more years of schooling would have a higher WTP for reduced pesticide residues in food [32]. Environmental economic theory assumes that the demand for health or environmental benefits should increase with income [33]. Consequently, high-income farmers are expected to be more willing to pay for the health risk reduction program. The positive sign of income in this study suggests that richer farmers are more likely to pay than lower income farmers, in agreement with the economic theory [34]. Zheng et al. (2015) also found that farmers’ farming income has a positive effect on their WTP for bio-pesticides [35]. 

The coefficient on respondents’ household size is negative and significant. These results imply that a respondent with a larger family would have a lower WTP because a larger family may have higher running costs and consequently lower WTP for health risk reductions [36].

The regression results show that three social capital variables have a statistically significant effect on farmers’ WTP (Table 4). The coefficients on social trust and social networks are significantly positive, implying that respondents with higher levels of social trust or greater involvement in social networks are more willingness to pay. These results are expected and understandable. Social trust is regarded as one of the most important components of social capital with significant influence on people’s WTP [21]. Pretty (2003) argue that social trust can influence an individual behavior because she or he expects that fellow citizens in the community will act in a similar way to protect the common good [20]. Social networks are mainly linked to farmers’ activation in collective activities and they are responsible for the flow of information on the health and environmental issues. People are more interested in collective issues are expected to be more willing to pay compared to individuals who are not active in social networks [22]. The variable of social reciprocity is negative and significant, indicating that respondents who thought people in their village can help each other would have a lower WTP for reducing the health risks related to pesticide use. One possible reason for this could be that respondents believed that other villagers would help them in case of they get diseases because of pesticide use. The coefficient on institutional trust is positive but insignificant.

Based on the estimation results, the mean WTP of the respondents is estimated to be 451.11 CNY (65.38 USD) per household per year, approximately equivalent to 0.94% of the average household income. Although currently there are no similar studies on pesticide use in China with which to compare this result, the mean WTP result of this study is comparable with other studies in developing countries. For example, farmers in the Philippines are willing to pay 13.5–20.5 USD per cropping season for avoiding health risks of pesticides [37]. Gallardo and Wang reported that apple growers’ and pear growers’ WTP were USD 26.03 per acre and USD 40.06 per acre, respectively, for reducing the probability of the toxicity of pesticides to natural enemies [38].

## 4. Conclusions

This study was aimed to estimate the willingness to pay of local farmers for reducing health risks associated with pesticide use and to explore the factors that influence individuals’ valuations by using the CVM in China. Our results indicate that there is a demand for reducing pesticide health risks. The mean WTP was estimated to be 451.11 CNY (65.38 USD) per household per year, which is equivalent to 0.94% of the average household income.

Several factors were investigated as explanatory variables for farmers’ monetary valuations on health risk reductions of pesticide use. The WTP is positively related to respondents’ education, farming income and risk perceptions, as expected. Farmers’ WTP is negatively related to household size. Based on these findings, there is a need to launch education or training programs for farmers to enhance their knowledge of pesticides and their risk perceptions. Information about health costs of pesticide use can serve as a basis for government decision-making for investments in rural health infrastructure [10,39].

Our results indicate that social capital is an important parameter explaining WTP of farmers for health risk reductions of pesticide use. Farmers’ social networks, social trust and institutional trust had positive effects on farmers’ WTP. Therefore, an enforcement of social capital variables, including social trust, social networks and institutional trust, is essential.

## Figures and Tables

**Table 1:** Socioeconomic characteristics of respondents.

Variable	Definition	Mean	Std. Dev.	Min	Max
Gender	1 = male; 0 = female	0.69	0.46	0	1
Age	Age of the respondent	52	9	30	86
Edu	Years of schooling	7.36	2.82	0	11
Agrin	Household farming income (USD/year)	3617	4826	72	31369
Hhsize	Number of household members living together	3.79	1.36	1	8

**Table 2:** Reasons for saying “yes” to the WTP question.

Reasons	Response	
	No.	Percentage
It’s good for my health in the future	87	55.77
The program can reduce the morbidity of farmers	59	37.82
I understand the harmful effects of pesticides	7	4.49
Others	3	1.92

**Table 3:** Reasons for saying “no” to the WTP question.

Reasons	Response	
	No.	Percentage
My household cannot afford it	39	38.61
The cost should be paid by local governments	36	35.64
I don’t think I will get sick because of pesticides	19	18.81
I don’t believe the program can reduce the morbidity of farmers	5	4.95
Others	2	1.98

**Table 4:** Estimated coefficients of logit model.

Variable	Definition	Estimated Coef. (Std. Err.)	Z-Scores
Bid	The bid used	−0.004 (0.0006) ***	−7.02
Risk	Risk perceptions (1 = extremely low; 2 = low; 3 = moderate; 4 = high; 5 = extremely high)	0.36 (0.18) **	2.03
Education	Years of schooling	0.11 (0.06) **	2.07
Income	Household farming income	0.34 (0.14) **	2.44
Hhize	Number of household members	−0.20 (0.11) *	−1.77
Network	Social networks	0.28 (0.17) *	1.68
Reciprocity	Social reciprocity	−0.49 (0.28) *	−1.79
Trust	Social trust	0.49 (0.25) **	1.96
Institution	Institutional trust	0.18 (0.18)	1.03
Constant	Constant term	0.84 (1.89)	0.45
Summary statistics			
Pseudo R squared = 0.26			
LR chi squared (9) = 87.21 ***			
Correctly classified = 77.87%			

* = significant at *p* < 0.1; ** = significant at *p* < 0.05; *** = significant at *p* < 0.01.

## References

[1] Carvalho F.P. (2006). Agriculture, pesticides, food security and food safety. Environ. Sci. Policy.

[2] Rahman S. (2013). Pesticide consumption and productivity and the potential of IPM in Bangladesh. Sci. Total Environ..

[3] Peshin R., Dhawan A.K. (2009). Integrated Pest Management: Dissemination and Impact. Am. J. Surg..

[4] Damalas C.A. (2009). Understanding benefits and risks of pesticide use. Sci. Res. Essays.

[5] Hvistendahl M. (2013). In rural Asia, locking up poisons to prevent suicides. Science.

[6] Verger P.J.P., Boobis A.R. (2013). Reevaluate Pesticides for Food Security and Safety. Science.

[7] Gress S., Lemoine S., Séralini G.E., Puddu P.E. (2015). Glyphosate-based Herbicides Potently affect cardiovascular system in mammals: Review of the literature. Cardiovasc. Toxicol..

[8] Mostafalou S., Abdollahi M. (2013). Pesticides and human chronic diseases, evidences, mechanisms, and perspectives. Toxicol. Appl. Pharm..

[9] Khan M., Damalas C.A. (2015). Farmers’ willingness to pay for less health risks by pesticide use: A case study from the cotton belt of Punjab, Pakistan. Sci. Total Environ..

[10] Garming H., Waibel H. (2009). Pesticides and farmer health in Nicaragua: A willingness-to-pay approach to evaluation. Eur. J. Health Econ..

[11] Wang H., Wang Y. (2017). Factors Influencing Indigenous Rice Protection in the Yuanyang Terraced Rice Fields of China. J. Resour. Ecol..

[12] Jin J., Wang W., He R., Gong H. (2017). Pesticide Use and Risk Perceptions among Small-Scale Farmers in Anqiu County, China. Int. J. Environ. Res. Public Health.

[13] Travisi C.M., Nijkamp P., Vindigni G. (2006). Pesticide risk valuation in empirical economics: A comparative approach. Ecol. Econ..

[14] Travisi C.M., Nijkamp P. (2008). Valuing environmental and health risk in agriculture: A choice experiment approach to pesticides in Italy. Ecol. Econ..

[15] Andersson H., Hole A.R., Svensson M. (2016). Valuation of small and multiple health risks: A critical analysis of SP data applied to food and water safety. J. Environ. Econ. Manag..

[16] Bateman I.J., Carson R.T., Brett D., Michael H., Nick H., Hett T., Jones-Lee M., Loomes G., Mourato S., Ozdemiroglu E. (2002). Economic Valuation with Stated Preference Techniques: A Manual.

[17] Yao R.T., Scarpa R., Turner J.A., Barnard T.D., Rose J.M., Palma J.H.N., Harrison D.R. (2014). Valuing Biodiversity enhancement in New Zealand’s planted forests: Socioeconomic and spatial determinants of willingness-to-pay. Ecol. Econ..

[18] Arega T., Tadesse T. (2017). Household willingness to pay for green electricity in urban and peri-urban Tigray, northern Ethiopia: Determinants and welfare effects. Energy Policy.

[19] Grootaert C., Narayan D., Jones V.N., Woolcock M. (2004). Measuring Social Capital: An Integrated Questionnaire.

[20] Pretty J. (2004). Social capital and the collective management of resources. Science.

[21] Jones N., Evangelinos K., Iosifides T., Halvadakis C.P., Sophoulis C.M. (2010). Social factors influencing perceptions and willingness to pay for a market-based policy aiming on solid waste management. Resour. Conserv. Recycl..

[22] Polyzou E., Jones N. (2011). Willingness to pay for drinking water quality improvement and the influence of social capital. J. Socio-Econ..

[23] Krupnick A., Alberini A., Cropper M., Simon N., O’Brien B., Goeree R., Heintzelman M. (2002). Age, health, and the willingness to pay for mortality risk reductions, a contingent valuation study of Ontario residents. J. Risk Uncertain..

[24] Vassanadumrongdee S., Matsuoka S. (2005). Risk Perceptions and Value of a Statistical Life for Air Pollution and Traffic Accidents, Evidence from Bangkok, Thailand. J. Risk Uncertain..

[25] Sandra H., Qin P., Krupnick A., Badrakh B., Batbaatar S., Altangerel E., Sereeter L. (2012). The willingness to pay for mortality risk reductions in Mongolia. Resour. Energy Econ..

[26] Palis F.G., Flor R.J., Warburton H., Hossain M. (2006). Our farmers at risk, behaviour and belief system in pesticide safety. J. Public Health.

[27] Lee C.-K. (1997). Valuation of nature-based tourism resources using dichotomous choice contingent valuation method. Tourism Manag..

[28] Hanemann W.M. (1994). Valuing the environment through contingent valuation. J. Econ. Perspect..

[29] Giraud K., Turcin B., Loomis J., Cooper J. (2002). Economic benefit of the protection program for the Steller sea lion. Mar. Policy.

[30] Mitchell R.C., Carson R.T. (1989). Using Survey to Value Public Goods: The Contingent Valuation Method.

[31] Louviere J.J., Hensher D.A., Joffre D.S. (2001). Stated Choice Methods: Analysis and Application.

[32] Wohl R.J.B., Vanhoehn E.O., Hoehn J. (1995). The Effect of Ambiguity on Willingness to Pay for Reduced Pesticide Residues. Antimicrob. Agents Chemother..

[33] Freeman A.M. (2003). The Measurement of Environmental and Resource Values: Theory and Methods.

[34] Chen W.Y. (2015). Public willingness-to-pay for conserving urban heritage trees in Guangzhou, South China. Urban For. Urban Green..

[35] Zheng S., Xu P., Lone T., Song S., Wang Z. (2015). Chinese Vegetable Producers’ Willingness to Pay for Bio-pesticides. J. Chin. Econ..

[36] Moffat B., Motlaleng G.R., Thukuza A. (2011). Households willingness to pay for improved water quality and reliability of supply in Chobe ward, Maun. Botsw. J. Econ..

[37] Cuyno L.C.M., Norton G.W., Rola A. (2001). Economic analysis of environmental benefits of integrated pest management: A Philippine case study. Agric. Econ..

[38] Gallardo R.K., Wang Q. (2013). Willingness to Pay for Pesticides’ Environmental Features and Social Desirability Bias: The Case of Apple and Pear Growers. J. Agric. Resour. Econ..

[39] Li P., Yan L., Pan S., Ma Y. (2017). Evaluation of Agricultural Ecological Security in Hubei Province. J. Resour. Ecol..

